# Vitamin D Deficiency: An Underestimated Factor in Sepsis?

**DOI:** 10.3390/ijms24032924

**Published:** 2023-02-02

**Authors:** Charlotte Delrue, Reinhart Speeckaert, Joris R. Delanghe, Marijn M. Speeckaert

**Affiliations:** 1 Department of Nephrology, Ghent University Hospital, 9000 Ghent, Belgium; 2 Department of Dermatology, Ghent University Hospital, 9000 Ghent, Belgium; 3 Department of Diagnostic Sciences, Ghent University, 9000 Ghent, Belgium; 4 Research Foundation-Flanders (FWO), 1000 Brussels, Belgium

**Keywords:** vitamin D deficiency, sepsis, mortality, vitamin D treatment

## Abstract

Vitamin D is an important immune modulator that is linked to infection susceptibility. It has been suggested that vitamin D deficiency plays a role in sepsis and septic shock because vitamin-D-related pathways are associated with various immunological, endocrine, and endothelial functions. Previous research has yielded inconclusive results regarding the link between mortality and vitamin D deficiency in sepsis patients. In patients with sepsis and severe vitamin D deficiency, an adequate vitamin D concentration may reduce mortality. Randomized controlled trials to assess the influence of vitamin D supplementation on clinical outcomes in sepsis patients with vitamin D deficiency are uncommon. We will provide an overview of the current knowledge about the relationship between vitamin D and sepsis in this review, as well as consider the potential value of vitamin D supplementation in this situation.

## 1. Introduction

**Figure 1:**
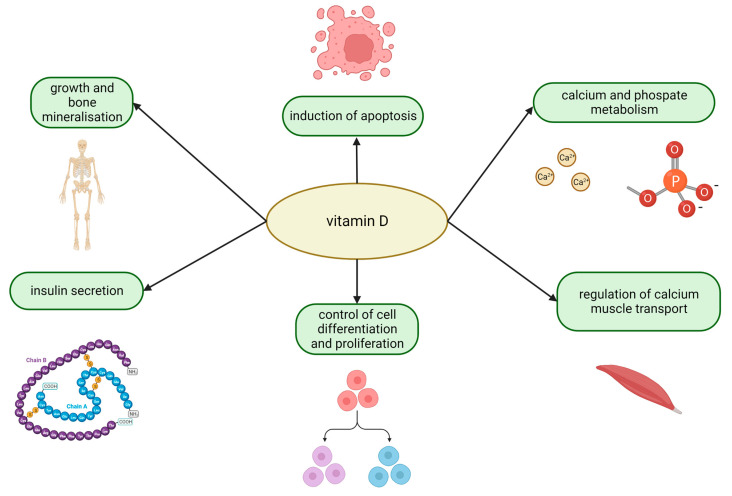
The major biological functions of vitamin D. Vitamin D is a multifunctional protein with antiproliferative and immunomodulating activities. Besides its well-known role in growth and bone mineralization, as well as in calcium an phosphate metabolism, vitamin D regulates apoptosis and controls cell differentiation and proliferation. Vitamin D is a fundamental component that controls the transport of calcium in muscles and is required for healthy insulin secretion.

Vitamin D is an essential nutrient for humans that was discovered in 1921. In addition to regulating calcium and phosphate metabolism, it is important for immunity, endothelial function, and antimicrobial activity (Figure 1) [1]. Since many immune system cells, both innate and adaptive, express vitamin D receptors (VDRs), vitamin D is crucial for immune system regulation [2]. Vitamin D deficiency is considered a global public health issue in Europe and the United States. Race, age, genotype, and the amount of vitamin D binding protein are all linked to it [3]. When admitted to the intensive care unit (ICU), a sizable percentage of critically ill patients (between 50% and 90%) have low 25-hydroxyvitamin D [25(OH)D] levels (ICU) [4,5,6,7]. Most studies have found a significant link between low vitamin D levels and disease severity, mortality, or a shorter time spent surviving in an ICU [6]. Some, but not all, studies that examined other clinically significant outcomes discovered that low vitamin D status was associated with lengthier ICU or hospital stays and a higher risk of acute kidney injury (AKI) (AKI) [5] and sepsis [8].

Sepsis was first described as a systemic inflammatory response brought on by infection in 1991. It was classified as severe sepsis when combined with organ dysfunction [9]. Sepsis is a potentially fatal illness that has high rates of morbidity and mortality [10]. The medical emergency of septic shock, which affects 15% of sepsis cases and accounts for 10% of ICU admissions, has a mortality rate of more than 50% [11]. Current recommendations for the management of sepsis and septic shock include the use of protocol-driven resuscitation care bundle therapy and an immediate assessment. The treatment plan includes fluid resuscitation, blood cultures, and the administration of broad-spectrum antibiotics [12]. Patients with sepsis who are vitamin D deficient are more likely to develop secondary infections [13]. Although sepsis patients frequently lack vitamin D, there is scant and conflicting evidence connecting vitamin D deficiency to sepsis mortality [1]. We will summarize the state of the science on the relationship between vitamin D and sepsis in this review and discuss the potential benefits of vitamin D supplementation in this circumstance.

## 2. The Role of Vitamin D and Sepsis

Since the first report by Lee et al. [6], a high prevalence of low vitamin D levels in both pediatric and adult critically ill patients has been confirmed. A lack of 25(OH)D reduces immune function, alters hormone metabolism, and increases the incidence of various viral and bacterial infections and critical illnesses, all of which increase mortality [14]. Uncertainty exists regarding the exact mechanisms by which a vitamin D deficiency raises the risk of sepsis and mortality. Although a number of puzzling factors, including renal excretion of vitamin D metabolites, comorbid conditions, pH, and nutritional supplementation, may all have an impact on the vitamin D status in critical illness, there is conflicting evidence regarding the potential impact of vitamin D on acute illness and its short- and long-term outcomes [15].

**Figure 2:**
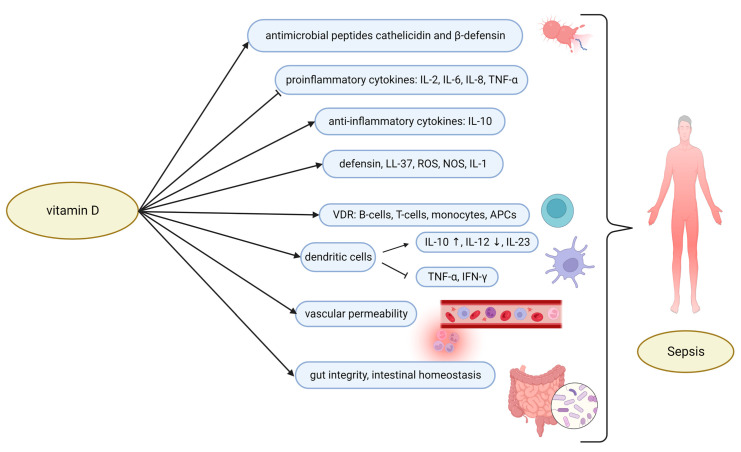
Overview of vitamin-D-mediated pathways in sepsis patients. Vitamin D exerts its immunomodulatory effects, e.g., through the production of antimicrobial peptide, the inhibition of proinflammatory cytokines, and the stimulation of anti-inflammatory cytokines. 1,25(OH)_2_D can prevent dendritic cells from maturing and differentiating by interacting with VDR. Vitamin D may have additional benefits in sepsis, such as effects on endothelial function, coagulation, and hemodynamic stability. Abbreviations: 1,25(OH)_2_D, 1,25-dihydroxyvitamin D; IL, interleukin; TNF-α, tumor necrosis factor-alpha; ROS, reactive oxygen species; NOS, nitric oxide synthase; VDR, vitamin D receptor; APCs, antigen-presenting cells; IFN-γ, interferon gamma.

Cathelicidin and β-defensin, which have a variety of immune system effector functions, are antimicrobial peptides that are produced as a result of vitamin D induction (Figure 2) [16,17]. They serve as a crucial link between the activation of Toll-like receptors (TLRs) and antibacterial reactions [18]. When vitamin D levels are low, the TLR stimulation of human macrophages results in a number of significant reactions, including (1) increased expression of VDR [17], (2) conversion of 25(OH)D to its most biologically active form, 1,25-dihydroxyvitamin D [1,25(OH)_2_D] [19], and (3) increased production of LL-37 (composed of 37 amino acids and two leucines at its N-terminal), the only cathelicidin-derived antimicrobial peptide found in humans [20,21].

Vitamin D can control the equilibrium between inflammation and tissue injury by promoting lymphocyte differentiation and development [22]. Vitamin D inhibits the production of pro-inflammatory cytokines such as interleukin (IL)-2, IL-6, IL-8, and tumor necrosis factor-alpha (TNF-α), and increases the production of anti-inflammatory cytokines such as IL-10 [2,23,24,25]. Immune cell gene expression is boosted by vitamin D [17] and a variety of inflammatory factors, such as defensin, LL-37, reactive oxygen species (ROS), nitric oxide synthase (NOS), and interleukin-1 (IL-1), which are all stimulated [20,21].

Numerous immune-regulating effects of vitamin D are carried out by VDR. Most immune cells, including monocytes, antigen-presenting cells (APCs) such as macrophages and dendritic cells, B cells, and T cells express VDR [26]. By increasing Th2 cytokine expression while suppressing Th1 cytokine, vitamin D encourages a switch in immune response from T helper (Th1) and Th17 to Th2 and regulatory T cells (Tregs). An uncontested proinflammatory state is suppressed as a result, and this may even contribute to the prevention of autoimmune diseases. People who are vitamin D deficient also exhibit this role in modulating inflammation, as evidenced by a decline in CD4/CD8 ratios, a sign of immune activation [27,28,29].

1,25(OH)_2_D can prevent dendritic cells from maturing and differentiating by interacting with VDR [30]. Additionally, it has the ability to control the expression and secretion of cytokines and chemokines by dendritic cells derived from monocytes, such as by promoting IL-10, IL-12, and IL-23 secretion and inhibiting tumor necrosis factor-alpha (TNF-α) and interferon-gamma (IFN-γ) secretion. More importantly, 1,25(OH)_2_D can indirectly inhibit B cell function by altering the response of CD4 T lymphocytes and inhibiting monocyte/macrophage cytokine secretion, resulting in an anti-infection effect for curing inflammation [14]. Additionally, since the renin–angiotensin–aldosterone system is activated by VDRs in the myocardium, any disruption in their function could result in cardiac hypertrophy and heart failure. VDRs are linked to both systolic and diastolic blood pressure [31].

The additional advantages of 25(OH)D and 1,25(OH)_2_D in sepsis include effects on coagulation, endothelial function, and hemodynamic stability [32,33]. Vitamin D can influence vascular permeability through a variety of genomic and extra-genomic pathways. 1,25(OH)_2_D is a transcriptional factor for endothelial NOS, capable of increasing gene expression and thus increasing nitric oxide production [34]. Vitamin D has also been shown to improve gut integrity and intestinal homeostasis by alleviating intestinal damage caused by bacterial lipopolysaccharide (LPS). The expression of epithelial membrane junction proteins can also be increased by vitamin D, which is beneficial when dealing with bacterial translocation events [35].

## 3. Neonates and Children

### 3.1. Vitamin D Deficiency

The prevalence of vitamin D deficiency ranges from 30% to 70% in the majority of pediatric intensive care units (PICUs) worldwide [36] (Table 1). Preterm babies are more likely to experience vitamin D deficiency than term ones [37,38]. On the other hand, sepsis mortality in children admitted to the PICU could reach 21.9% [39]. The first meta-analysis, which included 13 studies, found that children with sepsis had lower vitamin D levels than control subjects, and that the link between vitamin D deficiency (25(OH)D < 50 nmol/L or 20 ng/mL in critically ill patients and 25–30 nmol/L with severe deficiency) and sepsis was significant (odds ratio (OR): 1.13, 95% confidence interval (CI): 1.18–1.50, *p* < 0.05). This association persisted even after the analysis was stratified based on study design, sepsis diagnostic criteria, study site/medical conditions, the existence or absence of comorbidities, 25(OH)D assay techniques, and various age groups. The relationship between lower 25(OH)D levels and sepsis was unaffected by study location/medical conditions, diagnostic criteria for sepsis, and 25(OH)D assay methods [40]. The HPLC method [41,42] revealed a stronger link between vitamin D deficiency and sepsis in children than ELISA/other methods. When 25(OH)D levels were measured using ELISA, the association might have been underestimated [43,44,45,46]. Children (1 month–18 years old) showed a stronger association than neonates. Because the immune system is immature in newborns, the age at which sepsis is diagnosed may influence the association between vitamin D deficiency and sepsis. The included observational studies did not provide evidence that a lower 25(OH)D concentration preceded sepsis, so the causality of this association could not be established [40].

In a systematic review and meta-analysis, with a total of 27 studies (17 case–control studies and 10 cohort studies), the vitamin D status was investigated in critically ill children with sepsis. In the case–control studies, the sepsis group’s maternal and neonatal 25(OH)D concentrations were noticeably lower than those of the non-sepsis group (*p* < 0.001). In comparison to the non-sepsis group, the sepsis group had a significantly higher percentage of severe vitamin D deficiency (several different definitions: <5, <10, <11, <12 ng/mL, OR: 2.66, 95% CI: 1.13–6.25, *p* < 0.001). In the cohort studies, the incidence of sepsis was 30.4% in the group with lower 25(OH)D levels and 18.2% in the group with higher 25(OH)D levels. The rate of mechanical ventilation and 30-day mortality did not, however, differ in a statistically significant way. Because the 25(OH)D cut-off level in all studies was 20 ng/mL, which is higher than the definition of severe deficiency, the sepsis rate increased in the group with higher 25(OH)D levels [14]. Although an increased susceptibility to sepsis (OR: 2.65, 95% CI: 1.30–5.41) and the need for ventilator support (OR: 1.35, 95% CI: 1.03–1.77) was only found in critical care settings of the most developed countries, vitamin D deficiency (<50 nmol/L) was not associated with increased mortality in another meta-analysis (18 studies involving 2987 critically ill children) [47]. The outcome of this review’s update is consistent with the systematic review and meta-analysis conducted by McNally et al. [48].

In another meta-analysis based on 16 studies (2382 children at baseline with 1229 being vitamin D deficient (<50 nmol/L or 20 ng/mL)), vitamin-D-deficient children had a significantly higher risk of sepsis (OR: 2.35, 95% CI: 1.19–4.63, *p* = 0.01), a significantly higher pediatric risk of mortality III score (OR: 2.19, 95% CI: 1.13–4.25, *p* = 0.02), a longer length of hospital stay (OR: 4.26, 95% CI: 0.81–7.70, *p* = 0.02), and a higher duration of mechanical ventilation (OR: 1.89, 95% CI: 0.22–3.56, *p* = 0.03). On the other hand, there were no appreciable differences in the need for ventilation support between children with and without vitamin D deficiency (OR: 2.00, 95% CI: 0.98–4.07, *p* = 0.06), with relatively higher results in vitamin-D-deficient children [15]. Su et al. concentrated on vitamin D in critically ill children who were sepsis- and mortality-prone. In a meta-analysis with 23 studies with 4451 children, 2500 children with vitamin D deficiency (25(OH)D ≤ 50 nmol/L or 20 ng/mL) were identified. Children with low vitamin D concentrations (OR: 2.24, 95% CI: 1.42–3.53) had a higher risk of sepsis than children with adequate vitamin D levels. Additionally, but not as much as those with sepsis, children with vitamin D deficiency had higher acute and critical mortality (OR: 1.77, 95% CI: 1.26–2.49) than children with normal vitamin D levels. The severity of acute and critically fatal mortality was outweighed by the impact of increased sepsis. In all populations, there were children who lacked adequate amounts of vitamin D. Acute and critical care unit mortality and sepsis may both increase by up to 2.24-fold in children who are vitamin D deficient, according to this meta-analysis [49]. The prevalence of vitamin D deficiency (25(OH)D ≤ 50 nmol/L or 20 ng/mL) in children with sepsis was 64.0% in a systematic review examining the significance of vitamin D in acute and critically ill children (18 studies, 889 individuals) (95% CI: 52.0–74.4%, I^2^ = 89.3%, *p* < 0.0001). Children with low levels of 25(OH)D had a higher risk of dying, according to a meta-analysis on the link between vitamin D deficiency and mortality (18 cohort studies, 2463 people overall) (OR: 1.81, 95% CI: 1.24–2.64, *p* = 0.002, I^2^ = 25.7%, *p* = 0.153). Four (22.0%) of the eighteen studies statistically controlled for confounders. There were not enough studies on the mortality caused by sepsis and respiratory tract infections to perform a meta-analysis [50].

### 3.2. Vitamin D Supplementation in Neonatal Sepsis

Neonatal sepsis, a clinical syndrome, affects infants up to 28 days old and is characterized by systemic infection symptoms and the isolation of a bacterial pathogen from the bloodstream. Neonatal sepsis is divided into two categories: late-onset neonatal sepsis (LONS), which develops between 72 h and 28 days of age, and early-onset neonatal sepsis (EONS), which develops before 7 days of age but is sometimes restricted to the first 72 h of postnatal life. Neonatal septicemia continues to be one of the leading causes of mortality (it accounts for 30–50% of all neonatal deaths in developing countries) and morbidity, despite improvements in hygiene, the development of new and powerful antimicrobial agents for treatment, and advanced diagnostic techniques [57].

Vitamin D supplementation was assessed as an adjuvant therapy in neonates with sepsis in a randomized controlled trial (RCT). In this study, 60 neonates with sepsis were divided into two groups at random: group I received only antibiotic therapy, while group II received antibiotic therapy along with vitamin D (800 IU of vitamin D3 in a single oral dose for two weeks, from the diagnosis of sepsis until discharge). As a control group, 30 healthy neonates were included in this study. After 3, 7, and 10 days of treatment, there were significant differences in sepsis score and highly sensitive (hs)-CRP between groups I and II (*p* values for sepsis score were 0.009, 0.006, and 0.004, respectively, and for hs-CRP they were 0.015, 0.001, and 0.001, respectively). After 7 and 10 days of treatment, there was a discernible difference in the immature/total neutrophils (I/T) ratio (*p* values = 0.045, 0.025, respectively), but not after 3 days of treatment. Neonates with sepsis (groups I and II) had significantly lower serum 25(OH)D concentrations than controls (*p* < 0.05). In group II, there was a highly significant negative correlation between hs-CRP and serum 25(OH)D at baseline (r = −0.832 and *p* = 0.001) and after two weeks (r = −0.590 and *p* = 0.021) [51]. This was consistent with the significantly lower 25(OH)D concentrations found in mothers and full-term newborns who had sepsis, as well as the fact that there was a positive correlation between neonatal and maternal 25(OH)D concentrations [41]. According to the receiver operating characteristic (ROC) curve of specificity and sensitivity of 25(OH)D in the prediction of EONS, the sensitivity was 100%, the specificity was 73%, the positive predictive value was 73%, the negative predictive value was 100%, and the accuracy was 87% at a vitamin D cut-off value of 20 ng/mL [51]. Neonatal sepsis has been linked to cord blood 25(OH)D levels below 20 ng/mL because these levels are accompanied by altered monocyte responses that make newborns more vulnerable to infections [42]. Pre-treatment with 25(OH)D reversed the hyporesponsiveness that neonatal phagocytic cells exhibited in the presence of LPS, assisting in the overriding of endotoxin tolerance. The 1,25-dihydroxyvitamin D [1,25(OH)_2_D] action was mediated by the nicotinamide adenine dinucleotide phosphate oxidase system [44]. In contrast to these findings, Hagag et al. discovered that neonates with EONS had significantly higher pre-treatment 25(OH)D concentrations than the control group, as well as discovered a positive correlation between 25(OH)D and CRP, TNF-α, and IL-6 [51].

Fifty preterm infants with LONS and gestational ages of 28 weeks participated in a randomized, double-blind study and were randomly assigned to receive 400 or 800 IU of vitamin D per day. Both groups of infants had 76% vitamin D deficiency at enrollment, but only one infant in the 400 IU group and none in the 800 IU group still had a deficiency at 40 weeks postmenstrual age. At 40 weeks postmenstrual age, vitamin D concentrations were 54.8 ± 35.1 and 67.4 ± 37.1 ng/mL, respectively (*p* = 0.01) [52]. These results support the European Society of Pediatrics Gastroenterology and Nutrition’s (ESPGHAN) recommendation of 800 IU/day for vitamin D supplementation in preterm infants with sepsis, which differs from the American Academy of Pediatrics’ (AAP) recommendation of 400 IU/day [58,59]. At 1 week and after discharge, both groups’ serum levels of the pro-inflammatory cytokines IL-6 and TNF-α decreased without a difference in either group. There were no differences in the two groups’ anthropometric measurements, length of oxygen and respiratory support, duration of antimicrobial use, length of hospital stay, or mortality [52].

### 3.3. Vitamin D Supplementation in Children with Sepsis

An RCT was conducted in which 109 children with vitamin D deficiency and sepsis were randomly assigned to receive either 150,000 IU cholecalciferol or the placebo. The average vitamin D deficiency rate was 56.9%, with severe vitamin D deficiency accounting for 22.4%. Before treatment, the groups’ vitamin D concentrations were comparable. Both groups had significantly higher 25(OH)D concentrations 8 days after the intervention (*p* < 0.05). The treatment group’s vitamin D levels were, however, significantly higher than those of the control group following the intervention (*p* < 0.05). Vitamin D supplementation reduced IL-6, angiotensin-II (Ang-II), and TNF-α concentrations. This suggests that in the treatment group, systematic inflammation was effectively alleviated, and vitamin D prevented inflammation from exacerbating. As part of the renin–angiotensin system, Ang-II damages vascular endothelial cells by promoting liquid leakage, vasomotor dysfunction, and inflammatory cytokine release. Ang-II is regarded as a crucial indicator of conditions in sepsis patients because it plays significant roles in the onset of the condition. Vitamin D may increase blood flow and tissue perfusion by regulating Ang-II concentrations. The effects of vitamin D on septic shock and the differences between nutrients are also influenced by its effects on vascular tension. Furthermore, the treatment group had a lower cardiovascular sequential organ failure assessment (cv-SOFA) score (1.76 ± 0.8 vs. 2.3 ± 1.1) and a lower incidence of septic shock (7% vs. 20%). This suggests that vitamin D is required for controlling excessive inflammation, thereby preventing septic shock and protecting against this crisis [53]. Due to vitamin D’s potential to lessen an early blunted response to α-agonists and impaired endothelial reactivity to vasopressors, several clinical trials [54,55,56] have reported the impact of vitamin D deficiency on shock reversal (duration of vasopressor treatment or cumulative dosage). Changes in calcium homeostasis brought on by low vitamin D levels result in endothelial dysfunction and increased arterial stiffness [60,61]. Both groups’ mortality rates and ventilation duration were equal [56]. Weaning patients off ventilation is influenced by a variety of other factors, including cardiac function, primary disease, antibiotic use, and abdominal distension. No single intervention strategy can improve the prognosis of sepsis on its own [62].

## 4. Adults

### 4.1. Vitamin D Deficiency

Low serum 25(OH)D concentrations are a common issue in the US. Two thirds of the population had adequate levels of vitamin D, according to information from the US National Health and Nutrition Examination Survey (NHANES) between 2001 and 2006. The percentage of the population at risk of vitamin D deficiency was 25%, and it was 8% for vitamin D insufficiency [63]. The first meta-analysis investigated the association between low vitamin D concentrations (25(OH)D < 15–20 ng/mL) and the risk of sepsis. The pooled odds ratio for sepsis in participants with vitamin D deficiency was 1.78 (95% CI: 1.55–2.03, *p* < 0.01) compared to controls in studies that provided participant data, and it was 1.45 (95% CI: 1.26–1.66, *p* < 0.01) in studies that provided an adjusted odds ratio for developing sepsis [64]. In a critical care setting, including severe infection and sepsis, low serum 25(OH)D concentrations were associated with detrimental clinical outcomes, such as increased mortality, length of hospital stay, and AKI [5,65,66] (Table 2).

The association between adult patient mortality risk and serum 25(OH)D at admission was examined in a second meta-analysis. There were eight studies totaling 1736 patients. In patients with sepsis, lower serum 25(OH)D at admission was independently linked to an increased risk of mortality (adjusted relative risk (RR): 1.93, *p* < 0.001; I^2^ = 63%). While associations for vitamin D insufficiency (25(OH)D: 20–30 ng/mL) or deficiency (25(OH)D: 10–20 ng/mL) were not significant, patients with severe vitamin D deficiency (25(OH)D < 10 ng/mL) had a significantly higher mortality risk (adjusted RR: 1.92, *p* < 0.001). Further investigation revealed that the relationship between lower serum 25(OH)D levels and an increased mortality risk was constant across studies that used short-term (within 1 month) and long-term (3–2 months), prospective and retrospective, and different diagnostic criteria for sepsis (systemic inflammatory response syndrome, Sepsis-2.0, or Sepsis-3.0). These results suggest that sepsis patients may experience higher mortality rates if they have severe vitamin D deficiency (25(OH)D < 10 ng/mL). Large-scale prospective studies are necessary to verify these results [67].

### 4.2. Vitamin D Supplementation in Adults with Sepsis

Exogenous vitamin D supplementation was not linked to an increased 28-day cumulative survival rate, according to a small prospective randomized double-blind placebo study that included 57 sepsis patients, 20 patients with systemic inflammatory response syndrome (SIRS), and 20 healthy volunteers with normal physical examinations as controls [68].

In the correction of vitamin D deficiency in critically ill patients (VITdAL-ICU) study, a double-blind, randomized trial, 237 patients received vitamin D_3_, whereas 238 subjects were treated with a placebo [65]. Only half of the patients who received vitamin D3 treatment were able to achieve serum 25(OH)D concentrations > 30 ng/mL, despite using a high oral loading dose regimen with the intention of quickly restoring adequate 25(OH)D levels. This low number of vitamin D_3_ responders may be due to renal and drug-related impairments of the hepatic cytochrome P450 (CYP450) system, which is involved in vitamin D_3_ 25-hydroxylation, critical-illness-related compromised gastrointestinal function, and other factors [70,71]. In critically ill patients with vitamin D deficiency, high-dose vitamin D_3_ administration did not shorten hospital stays, decrease hospital mortality, or lower 6-month mortality compared to the placebo. The analysis of the causes of death showed that there were no differences between the vitamin D_3_ group and the placebo group in terms of the proportions of deaths from sepsis, cardiovascular, neurologic, and other causes. Hospital mortality was lower in the severe vitamin D deficiency subgroup (28.6% in the vitamin D_3_ group vs. 46.1% in the placebo group, HR: 0.56, 95% CI: 0.35–0.90, *p* = 0.04), but this finding should be considered to be hypothesis-generating and warrants further investigation. Vitamin D supplementation may reduce the occurrence of adverse outcomes in the ICU (e.g., nosocomial infections) [65].

An RCT examined the effects of cholecalciferol (200,000 IU (*n* = 10) vs. 400,000 IU (*n* = 10)) on changes in vitamin D status and cathelicidin (LL-37) levels in septic ICU patients treated with the placebo (*n* = 10) versus cholecalciferol. In patients with severe sepsis or septic shock, high-dose cholecalciferol supplementation improved 25(OH)D and bioavailable 25(OH)D levels quickly and safely. Changes in bioavailable 25(OH)D were linked to increases in circulating LL-37 levels [72]. LL-37 is a chemotactic agent for T cells, monocytes, and neutrophils [73], and works in conjunction with defensins to have synergistic antibacterial effects (another major class of endogenous, anti-microbial peptides) [74]. As a result, the innate immune system’s first line of defense may be strengthened by the expression of LL-37 by neutrophils and epithelial cells [75]. In contrast to these results, a different RCT found that giving 1,25(OH)_2_D to sepsis patients did not raise their plasma LL-37 levels [76]. However, it is crucial to remember that the intervention group in this study only received a single dose of 1,25(OH)_2_D, a substance with a brief half-life [77]. Because of this, 1,25(OH)_2_D significantly increased leukocyte LL-37 mRNA expression but did not significantly raise systemic levels of antimicrobial peptide [76]. Cholecalciferol supplementation also resulted in a significant reduction in the systemic levels of IL-1β and IL-6, but not of TNF-α. In the early inflammatory response that characterizes sepsis, both IL-1 and IL-6 play important roles [72].

Using data from the prospectively collected septic shock registry, an observational, single-center study was carried out in the emergency room of a tertiary referral academic facility in Seoul, South Korea. The study included 302 patients in total: 236 (78.1%) of them had vitamin D deficiency, which was significantly more common in non-survivors than in survivors (89.3% vs. 73.9%, *p* = 0.004). Patients with vitamin D deficiency died more frequently than patients without it (31.8% vs. 13.6%, *p* = 0.004). After adjusting for significant factors such as hemoglobin levels, malignancy, albumin levels, SOFA scores, and hyperlactatemia, vitamin D deficiency was found to be an independent predictor of 30-day mortality [1].

### 4.3. Vitamin D Supplementation in Patients with COVID-19

According to a recent study, vitamin D is essential for immune health and cellular resilience and its lack may cause cytokine storms in newly acquired coronavirus infections [78]. It could prevent acute respiratory distress syndrome (ARDS) by lowering pro-inflammatory Th1 cytokine synthesis, such as TNF-α and IFN-γ, while increasing anti-inflammatory cytokine expression in macrophages [79]. Vitamin D is a biological predictor of COVID-19 outcomes, according to growing pre-clinical and clinical evidence. By regulating the activity of the renin–angiotensin system and the expression of the angiotensin-2 converting enzyme (ACE2), which decreased pulmonary permeability in an ARDS experimental model, vitamin D may counteract the harmful effects of COVID-19. By binding to the vitamin D response elements (VDRE) found in gene promoters, ACE2 either activates or represses gene expression [80,81]. This is an important step because severe acute respiratory syndrome coronavirus-2 (SARS-CoV-2) has been shown to infect host cells by using ACE2 as a receptor [82], and to downregulate ACE2 expression [83,84]. A cytokine storm caused by the host’s downregulation of ACE2 during COVID-19 infection results in ARDS [85]. In COVID-19 patients, vitamin D supplementation may lessen cytokine storms by influencing the activity of the renin–angiotensin system and the production of ACE2. In a prospective RCT consisting of hospitalized adults infected with moderate-to-severe SARS-CoV-2 infection, vitamin D was administered orally (alfacalcidol 1 µg/day) to 85 patients and intramuscularly (cholecalciferol 200,000 IU) to another group of 85 subjects for a minimum of five days. Clinical improvement (45% versus 55%), sepsis occurrence (35% versus 65%), the length of hospital stay (8.6 versus 6.8 days), the need for high oxygen or non-invasive mechanical ventilation (67% versus 33%), the need for a mechanical ventilator (25% versus 75%), and the monitored laboratory parameters all favored high-dose vitamin D. High-dose vitamin D was linked to better clinical improvement and fewer negative outcomes when compared to low-dose vitamin D [69].

## 5. Conclusions

In patients with acute and critical care conditions, vitamin D remains an appealing biomarker and potential therapeutic agent. Vitamin D’s multiple functions in the immune system’s response to infection may make it an important component in the fight against sepsis. Due to the possibility that the underlying causes of vitamin D deficiency in these patients may also be related to the underlying causes of the critical disease, the relatively high prevalence of vitamin D deficiency in critically ill patients raises the possibility that it may play a prognostic role. The disparities between pediatric and adult populations in terms of the relationship between vitamin D deficiency and higher mortality may be due to differences in the quality and study populations of the individual studies included in the meta-analyses. Future research will require a reliable 25(OH)D assay technique and standardized sepsis diagnostic criteria, and the study site and medical conditions should be taken into consideration as confounding variables. It is well known that C-3 epimerization of the A ring of all vitamin D metabolites can result in falsely elevated blood levels when different methods for 25(OH)D assessment are used. C-3 epimers have been found in neonates, children, and adults, with neonates and children having the highest concentrations (up to 60%). Because C-3 epimers have lower biological activity and a lower influence on calcemia, methods used for 25(OH)D assessment, particularly in neonates and children, should have very low cross-reactivity (for example, <1%) with epimers.

Most obesity parameters, such as body mass index (BMI), total fat mass, subcutaneous and visceral adiposity, and waist circumference, are inversely related to 25(OH)D. These findings have been made not only in adults, but also in children and the elderly. Vitamin D sequestration or dilution in adipose tissue, increased vitamin D catabolism in adipose tissue, decreased 25-hydroxylation, and decreased sun exposure may result in low plasma 25(OH)D concentrations. On the other hand, low plasma 25(OH)D concentrations are linked to a high prevalence of obesity in children, adults, and elderly women, and low vitamin D intake may predict later obesity, metabolic syndrome, and even obesity onset. Moreover, obesity reduces the effect of vitamin D supplementation in obese patients, underscoring the critical need to develop strategies for optimal vitamin D supplementation in obese people. Given the positive relationship between obesity and vitamin D deficiency, as well as the increased risk of infection and inflammation in people with obesity, this could be a confounding or interacting factor in the relationship between vitamin D and sepsis.

Vitamin D metabolism may be affected by a number of drugs, whereas sepsis may also cause vitamin D deficiency. It is important to determine the type of association between vitamin D deficiency and sepsis, as well as the pediatric risk of mortality III score, the length of hospital stay, and the time spent on mechanical ventilation. In addition, it is important to thoroughly investigate the key molecular pathways that link vitamin D deficiency to a poor prognosis for sepsis patients. While adequate vitamin D before sepsis may be protective, vitamin D supplementation during sepsis may have no effect on the immune response to bacteria. Cholecalciferol may act as a substrate for the synthesis of 1,25(OH)_2_D, but there is no assurance that it will increase serum 1,25(OH)_2_D levels, which could limit its benefits. Many RCTs found no benefit in severe infections or sepsis after vitamin D normalization in vitamin-D-deficient patients. The reason for this could be a lack of power, low dosages, or a short duration of supplementation. More research is needed to determine whether routine vitamin D supplementation for optimizing vitamin D status in cases of septic shock is warranted to improve clinical outcomes. Vitamin D has been shown to reduce mortality rates in critically ill adults, whereas it is unclear whether it reduces mortality rates in critically ill children. More clinical trials should confirm these findings and determine the best vitamin D supplementation dose and method for critically ill children. Finally, vitamin D is commonly prescribed and also used by patients as an over-the-counter formulation. Patients on high doses of vitamin D can be at risk of vitamin D toxicity. To avoid prescription errors that result in negative outcomes, physicians should be aware of the importance of closely monitoring these supplements, particularly in populations at risk due to high dose requirements.

## Figures and Tables

**Table 1:** Overview of studies investigating vitamin D deficiency and vitamin D supplementation in neonates and children with sepsis.

	Study Design	Study Population	Method of Vitamin D Analysis	Major Findings	Ref.
Vitamin D deficiency	Prospective cohort study	316 critically ill children	Immunoassay	Hypovitaminosis D is common in critically ill children and linked to higher inotrope levels in the postoperative cardiac population, but not to PICU length of stay or hospital survival.	[36]
	Cross-sectional analysis	71 very preterm infants (< 32 weeks of gestational age) vs. 108 preterm infants (32–36 6/7 weeks of gestational age) vs. 292 term infants (≥ 37 weeks of gestational age)	CLIA	Infants born before 32 weeks gestation had 2.5 times higher odds of 25(OH)D levels < 20 ng/mL compared to more mature infants (95% CI: 1.2–5.3).	[37]
	Cohort study	120 preterm infants (67 infants born < 28 PMA and 53 infants born between 28–32 weeks PMA)	Liquid chromatography/tandem mass spectrometry	Mean serum 25(OH)D at birth was significantly lower in infants born < 28 weeks than at 28–32 weeks gestation (*p* = 0.02).	[38]
	Meta-analysis	13 studies including neonates and children (< 18 years) with sepsis		A significant relationship existed between vitamin D deficiency and sepsis with an OR of 1.13 (95% CI: 1.18–1.50, *p* < 0.05).	[40]
	Cross sectional study	50 infants with EONS in term infants vs. 50 HC	HPLC	The EONS group’s neonatal 25(OH)D levels were significantly lower than those of the control group (*p* < 0.001). Significantly lower 25(OH)D levels in mothers and full-term neonates with sepsis (*p* < 0.001), along with a positive correlation between neonatal and maternal 25(OH)D levels in both EONS patients and HC (r = 0.29, *p* = 0.04, and r = 0.58, *p* = 0.001, respectively).	[41]
	Case–control study	40 infants with EONS vs. 43 HC	HPLC	Low cord blood 25(OH)D level (< 30 ng/mL) was associated with a 5.6 times higher risk of EONS (OR: 5.6; 95% CI: 1.3–23.5).	[42]
	Cross-sectional study	124 critically ill children with sepsis aged 1–12 years vs. 338 HC	ELISA	When compared to HC, critically ill children with sepsis had a greater prevalence of vitamin D deficiency (50.8% vs. 40.2%, *p* = 0.04).	[43]
	Cross-sectional study	120 children with suspected sepsis vs. 30 HC	ELISA	Children with sepsis had a 51% lower level of 25(OH)D than HC (*p* < 0.001).	[44]
	Cross-sectional study	117 premature infants with gestational age < 67 weeks (severe vitamin D deficiency (group 1, *n* = 63, 25(OH)D < 5 ng/mL), vitamin D insufficiency (group 2, *n* = 24, 25(OH)D = 5–15 ng/mL), and vitamin D sufficiency (group 3, *n* = 13, 25(OH)D ≥ 15 ng/mL))	ELISA	The frequency of both EONS and LONS was not significantly linked to low cord blood 25(OH)D levels (5 ng/L).	[45]
	Prospective observational study	154 critically ill patients	ELISA	Regarding the severity of the illness at admission, the length of the PICU stay, the requirement for mechanical ventilation, the duration of ventilator support, septic shock, liver failure, and the requirement for renal replacement therapy, children with low vitamin D levels did not differ from children without low vitamin D levels.	[46]
	Systematic review and meta-analysis	27 studies (17 case–control studies and 10 cohort studies)		Case–control studies: the association between vitamin D deficiency and sepsis was significant with an OR of 2.66 (95% CI: 1.13–6.25, *p* < 0.001). Cohort studies: sepsis was more common in the low 25(OH)D group than in the higher 25(OH)D group (30.4% and 18.2%, respectively). Meta-analysis: vitamin D insufficiency was found to significantly increase the risk of sepsis (OR: 2.65, 95% CI: 1.3–5.41) and ventilation support necessary (OR: 1.35, 95% CI: 1.03–1.77) in extremely high-developed nations.	[47]
	Systematic review and meta-analysis	17 studies including a total of 2783 critically ill children		In wealthy countries, vitamin D deficiency was linked to increased mortality (OR: 1.62, 95% CI: 1.11–2.36), illness severity, and need for PICU interventions (cardiovascular support and mechanical ventilation).	[48]
	Meta-analysis	16 studies including 2382 children at baseline with 1229 being vitamin D deficient (< 50 nmol/L or 20 ng/mL)		Vitamin-D-deficient children had a significantly higher risk of sepsis (OR: 2.35, 95% CI: 1.19–4.63, *p* = 0.01), a significantly higher pediatric risk of mortality III score (OR: 2.19, 95% CI: 1.13–4.25, *p* = 0.02), a longer length of hospital stay (OR: 4.26, 95% CI: 0.81–7.70, *p* = 0.02), and a higher duration of mechanical ventilation (OR: 1.89, 95% CI: 0.22–3.56, *p* = 0.03). The need for ventilation support in vitamin-D-deficient children, on the other hand, did not differ significantly from non-vitamin-D-deficient children (OR: 2.00, 95% CI: 0.98–4.07, *p* = 0.06), with relatively higher results in vitamin-D-deficient children.	[15]
	Meta-analysis	23 studies including 4451 children (2500 children with vitamin D deficiency (25(OH)D ≤ 50 nmol/L or 20 ng/mL))		Children with low vitamin D levels had a greater risk of sepsis (OR: 2.24; 95 CI: 1.42–3.53) than children with adequate vitamin D levels. Additionally, compared to children with adequate vitamin D levels, children with vitamin D insufficiency had greater rates of acute and critical mortality (OR: 1.77; 95% CI: 1.26–2.49), though not as high as sepsis.	[49]
	Systematic review and meta-analysis	18 cohort studies including 2463 children hospitalized with acute or critical conditions		Children with 25(OH)D deficiencies had a 1.81 increased mortality risk (OR: 1.81; 95% CI: 1.24–2.64, *p* = 0.002).	[50]
Vitamin D supplementation neonatal sepsis	RCT	60 neonates with sepsis (group I: 30 neonates with sepsis treated with only antibiotics and group II: 30 neonates with sepsis treated with both antibiotics and vitamin D (800 IU of vitamin D_3_ in a single oral dose for 2 weeks)) and 30 HC	ELISA	After 3, 7, and 10 days of treatment, there were significant differences in sepsis score and hs-CRP between group I and II (*p* values for sepsis score were 0.009, 0.006, and 0.004, respectively, and for hs-CRP they were 0.015, 0.001, and 0.001, respectively). Significant negative correlation between hs-CRP and serum 25(OH)D levels in group II on entry and after 2 weeks of treatment (r = −0.832, *p* = 0.001, and r = −0.590, *p* = 0.021, respectively). ROC analysis showed that at a vitamin D cut-off value of 20 ng/mL, the sensitivity was 100%, the specificity was 73%, the positive predictive value was 73%, the negative predictive value was 100%, and the accuracy was 87% in predicting EOS.	[51]
	RCT	Preterm infants with gestational age ≥ 28 weeks with LONS. Subjects were randomly assigned to receive 400 or 800 IU/day of vitamin D_3_.	ELISA	At enrollment, 76% of the infants were vitamin-D-deficient in both groups, but only one infant in the 400 IU group and none in the 800 IU group remained deficient at 40 weeks postmenstrual age. Serum pro-inflammatory cytokine IL-6 and TNF-α concentrations decreased in both groups at 1 week and at discharge, with no difference between groups. After the intervention, vitamin D levels in the treatment group were significantly higher than in the control group (*p* < 0.05). Vitamin D supplementation reduced Ang-II, IL-6, and TNF-α concentrations (all *p* < 0.05).	[52]
Vitamin D supplementation in children with sepsis	RCT	109 children with vitamin D deficiency and sepsis randomly assigned to either 150,000 IU cholecalciferol or placebo	ELISA	The treatment group had a lower cv-SOFA score (1.76 ± 0.8 vs. 2.3 ± 1.1) and a lower incidence of septic shock (7% vs. 20%). There was no difference between the two groups in terms of ventilation time or fatality rates.	[53]
	Prospective observational cohort study	90 children		Vitamin D deficiency was significantly linked to vasopressor use (RR: 1.6, 95% CI: 1.2–2.3, *p* < 0.01), mechanical ventilation (RR: 2.2, 95% CI: 1.2–3.9, *p* < 0.01), septic shock (RR: 1.9, 95% CI: 1.3–2.9, *p* < 0.01), and fluid bolus > 40 mL/kg in the first 24 h of admission (RR: 1.5, 95% CI: 1.1–2.1, *p* < 0.05).	[54]
	Retrospective cohort study	107 patients with sepsis	CLIA	The 52 patients with pneumonia in the extremely low vitamin D group required mechanical ventilation for a longer period of time (9 days (3.75–12.5 days) versus 4 days (2–9 days), *p* < 0.04), and the 66 with septic shock required vasopressor support for a longer period of time (7 days (4–10 days) versus 4 days (2–7.25 days), *p* < 0.02).	[55]
	Prospective observational cohort study	54 children	ECL	Low vitamin levels were linked to higher PIM or SOFA scores (r = 0.29, *p* = 0.04, and r = 0.29, *p* < 0.05, respectively). Children who were mechanically ventilated had a median serum 25(OH)D level that was considerably lower than children who were not (*p* < 0.01).	[56]

Abbreviations: 25(OH)D, 25-hydroxyvitamin D; Ang-II, angiotensin II; CI, confidence interval; CLIA, chemiluminescence immunoassay; cv, cardiovascular; ECL, electrochemiluminescence; ELISA, enzyme-linked immunosorbent assay; EONS, early-onset neonatal sepsis; HC, healthy controls; HPLC, high-performance liquid chromatography; hs-CRP, high-sensitive; IL-6, interleukin-6; LONS, late-onset neonatal sepsis; OR, odds ratio; PICU, pediatric intensive care unit; PIM, pediatric index of mortality; PMA, postmenstrual age; RCT, randomized controlled trial; RR, relative risk; SOFA, sequential organ failure assessment; TNF-α, tumor necrosis factor-alpha.

**Table 2:** Overview of studies investigating vitamin D deficiency and vitamin D supplementation in adults with sepsis.

	Study Design	Study Population	Method of Vitamin D Analysis	Major Findings	Ref.
Vitamin D deficiency	Observational study	2399 patients, age ≥ 18 years		A lack of 25(OH)D prior to hospitalization is a significant predictor of both short- and long-term all-cause patient mortality as well as blood culture positivity in a population of critically ill patients.	[5]
	Systematic review and meta-analysis	10 observational studies		In studies that reported participant numbers, the pooled odds ratio of sepsis in participants with vitamin D deficiency was 1.78 (95% CI: 1.55–2.03, *p* < 0.01) compared to controls, and it was 1.45 (95% CI: 1.26–1.66, *p* < 0.01) in studies that reported an adjusted odds ratio of vitamin D deficiency for developing sepsis.	[64]
	Prospective observational study	258 patients	HPLC and tandem mass spectrometry	After adjusting for age, gender, race, and comorbidities (myocardial infarctions, acute renal failure, and pneumonia), severe and moderate vitamin D deficiency were related inversely to surgical intensive care unit length of stay (r = 0.194; *p* = 0.001), surgical intensive care unit treatment cost (r = 0.194; *p* = 0.001), and mortality (r = 0.125; *p* = 0.023), compared to the mildly vitamin-D-deficient group.	[66]
	Meta-analysis	8 studies with 1736 patients		Severe vitamin D deficiency may be associated with an increase in mortality in adult sepsis patients.	[67]
Vitamin D supplementation in adults with sepsis	RCT	475 patients (237 in the vitamin D_3_ group and 238 in the placebo group)	CLIA	High-dose vitamin D_3_ administration versus placebo did not reduce hospital length of stay, hospital mortality, or 6-month mortality in critically ill patients with vitamin D deficiency. The severe vitamin D deficiency subgroup had lower hospital mortality, but this finding should be considered in the generation of hypotheses and warrants further investigation.	[65]
	RCT	22 SIRS patients and 20 HC	ECL	Serum 25(OH)D3 levels in sepsis ICU patients were lower than in healthy individuals, but sepsis and SIRS patients did not differ significantly. Patients with sepsis had serum 25(OH)D3 levels that were correlated with gender, with female levels being lower than male levels, but not with age. Exogenous vitamin D_3_ supplementation had no positive impact on ICU sepsis patients’ prognoses. The APACHE II score and 25(OH)D3 20 g/L were risk factors for prognosis in ICU patients with sepsis.	[68]
	RCT	Placebo (*n* = 10) versus 200,000 IU cholecalciferol (*n* = 10) versus 400,000 IU cholecalciferol (*n* = 10), within 24 h of new-onset severe sepsis or septic shock	ELISA	In patients with severe sepsis or septic shock, high-dose cholecalciferol supplementation improves 25(OH)D and bioavailable 25(OH)D levels quickly and safely.	
Vitamin D supplementation in patients with COVID-19	RCT	116 patients (group 1, *n* = 58: orally administered alfacalcidol 1 µg/day; group 2, *n* = 58: intramuscularly administered cholecalciferol 200,000 IU)		When compared to low-dose vitamin D, high-dose vitamin D was associated with better clinical improvement and fewer negative outcomes, making it a promising treatment for suppressing cytokine storms in COVID-19 patients.	[69]

Abbreviations: 25(OH)D, 25-hyroxyvitamin D; APACHE, acute physiology and chronic health evaluation; CI, confidence interval; CLIA, chemiluminescence immunoassay; COVID-19, coronavirus disease 2019; ECL, electrochemiluminescence; ELISA, enzyme-linked immunosorbent assay; HPLC, high-performance liquid chromatography; ICU, intensive care unit; RCT, randomized controlled trial; SIRS, systemic inflammatory response syndrome.

## Data Availability

Not applicable.
